# 6PPDQ Exposure Exacerbates Seizure-Induced Neuronal Damage via the TP53/Nrf2 Axis: An Integrated Strategy Combining Network Toxicology and Experimental Validation

**DOI:** 10.3390/toxics14050443

**Published:** 2026-05-19

**Authors:** Ruijin Xie, Wei Xiao, Hua Xu, Yufan Luo, Xue Xiao, Qiyang Pan, Shengjie Xu, Li Liu, Chenyu Sun, Yueying Liu

**Affiliations:** 1 School of Normal Education, Yangzhou Polytechnic College, Yangzhou 225009, China; 2 School of Medicine, Jiangnan University, Wuxi 214122, China; 3 Department of Psychiatry and Psychology, Mayo Clinic, Rochester, MN 55905, USA; 4 Mayo Clinic School of Graduate Medical Education, Mayo Clinic College of Medicine and Science, Rochester, MN 55905, USA; 5 Department of Internal Medicine, The Second People’s Hospital of Hefei, Guangde Road, Hefei 230061, China; 6 Division of Public Health, Infectious Diseases, and Occupational Medicine, Mayo Clinic, Rochester, MN 55905, USA; 7 School of Public Health, University of Minnesota-Twin Cities, Minneapolis, MN 55455, USA

**Keywords:** seizure, neuronal damage, inflammation, 6PPDQ, Nrf2, TP53

## Abstract

As an emerging tire wear-derived environmental contaminant, 6PPD-quinone (6PPDQ) has raised significant concerns regarding its neurotoxic potential, particularly for children exposed to recycled tire crumb rubber in playgrounds. However, the molecular mechanisms by which 6PPDQ influences neurological disorders such as epilepsy remain poorly understood. In this study, we employed an integrative framework combining network toxicology, bulk analysis of human epileptic brain tissues, Mendelian randomization, and molecular dynamics simulations to elucidate these mechanisms. Our findings, validated through CETSA-WB and SPR, identify 6PPDQ as a direct ligand that binds to and stabilizes neuronal TP53. Through a synergistic double-hit mechanism, 6PPDQ directly engages the TP53 pathway while simultaneously triggering microglial interleukin-6 secretion. These converging pathways lead to the suppression of the master antioxidant regulator Nrf2, resulting in glutathione depletion, excessive reactive oxygen species accumulation, and exacerbated neuronal damage under excitotoxic stress. Experimental validation using glutamate-induced HT22 cell models and microglia–neuron crosstalk systems confirmed that targeting the TP53/Nrf2 axis or scavenging ROS significantly attenuates 6PPDQ-induced neurotoxicity. Our findings highlight critical risks to pediatric neurological health posed by tire-derived contaminants and identify the TP53/Nrf2 axis as a promising therapeutic target. Furthermore, this work provides a robust scientific basis for refining risk assessment frameworks and developing regulatory strategies to mitigate environmental exposure to 6PPDQ.

## 1. Introduction

With rapid global industrialization, environmental pollution originating from manufacturing processes has become increasingly pervasive, raising significant concerns about its neurotoxic potential. Recently, 6PPD-quinone (6PPDQ), a transformation product of the tire antioxidant 6PPD, has emerged as a widespread environmental contaminant. First identified as the causative agent of mass mortality events in Pacific Northwest Coho salmon, 6PPDQ is generated when 6PPD on tire surfaces reacts with atmospheric ozone [1,2]. It subsequently infiltrates aquatic ecosystems and urban environments through stormwater runoff. Recent proposals for freshwater quality criteria suggest short-term and long-term benchmarks of 0.20 μg/L and 0.15 μg/L, respectively. Notably, 6PPDQ has been detected in playgrounds surfaced with recycled tire crumb rubber, establishing a direct exposure route for children via inhalation and incidental ingestion [3,4].

The pediatric population exhibits unique vulnerability to environmental pollutants due to the high plasticity of the developing brain and the relative immaturity of the blood–brain barrier (BBB) [5,6]. Although the acute neurotoxicity of 6PPDQ has been documented in aquatic species, its impact on chronic neurological disorders, such as epilepsy, remains poorly characterized. Seizures, defined by hypersynchronous neuronal discharges, frequently lead to cumulative neuronal damage driven by oxidative stress and neuroinflammation [7,8]. In this context, we hypothesized that 6PPDQ exposure may exacerbate seizure-induced neuronal injury through a synergistic “double-hit” mechanism. First, a mechanistic convergence likely exists between 6PPDQ toxicity and epileptic pathology. As a potent environmental pro-oxidant, 6PPDQ induces excessive accumulation of reactive oxygen species (ROS). Similarly, epileptic seizures are associated with heightened neuronal activity, excessive energy consumption, and substantial endogenous ROS production [7,8]. We therefore postulated that pre-existing oxidative stress triggered by 6PPDQ exposure may overwhelm neuronal antioxidant defense systems, thereby amplifying seizure-associated oxidative damage [9]. Second, the populations at risk substantially overlap. Children are frequently exposed to 6PPDQ through recycled tire-derived playground materials, while simultaneously exhibiting increased susceptibility to seizures due to the immaturity of the BBB and the heightened vulnerability of the developing brain. Collectively, these observations suggest that 6PPDQ may act as a critical environmental risk modifier that lowers seizure thresholds and aggravates neuronal injury through disruption of redox homeostasis.

In this study, we employed an integrative framework combining network pharmacology, computational docking, molecular dynamics simulations, and transcriptomic RNA-sequencing to elucidate the mechanisms by which 6PPDQ influences neuronal health. Our analysis identified Interleukin-6 (IL-6) and the TP53/Nrf2 axis as pivotal mediators of the 6PPDQ-induced response. We hypothesize that 6PPDQ exposure facilitates the excessive accumulation of reactive oxygen species (ROS), thereby aggravating neuronal damage. Through in vitro experimental validation, we confirmed the regulatory roles of the Nrf2 pathway and TP53 in modulating ROS homeostasis. Collectively, our findings demonstrate that 6PPDQ acts as a potent environmental stressor that exacerbates epilepsy-related neuronal injury, providing a mechanistic basis for assessing the risks of tire-derived contaminants to pediatric neurological health.

## 2. Materials and Methods

### 2.1. Network Toxicology Analysis

To systematically identify potential human gene targets that mediate the link between 6PPDQ-induced neurotoxicity and seizure susceptibility, an integrated network toxicology approach was adopted, which was modified from well-established analytical frameworks in our previous research [10,11]. The entire analytical workflow was performed in the following sequential steps:

(1) Acquisition of 6PPDQ-associated gene targets: Human genes potentially modulated by 6PPDQ were retrieved from the Comparative Toxicogenomics Database (CTD, http://ctdbase.org/), a manually curated cross-species resource that comprehensively integrates chemical–gene interactions, chemical–disease associations, and disease–gene regulatory relationships. The keyword “6PPDQ” was used as the retrieval query to extract all relevant gene entries with experimentally documented or in silico predicted interactions with 6PPDQ.

(2) Collection of seizure-related gene targets: Genes associated with seizure disorders were obtained from the GeneCards database (https://www.genecards.org/), a comprehensive and integrative compendium of human gene annotations that curates genomic, transcriptomic, proteomic, and functional gene data from more than 200 integrated web sources. The search was conducted with the keyword “Seizures”, and to prioritize biologically relevant candidates and eliminate background noise from weakly associated gene entries, only genes with a Relevance Score ≥1.0 were retained for subsequent downstream analyses.

(3) Intersection analysis of gene sets: Overlapping genes between the 6PPDQ-modulated gene dataset and the seizure-associated gene dataset were identified via Venn diagram analysis using the web-based tool InteractiVenn (http://www.interactivenn.net/). This step yielded a core set of candidate genes that may potentially mediate the neurotoxic effects of 6PPDQ in the pathophysiological process of seizures. These intersecting genes were designated as the foundational gene set for all subsequent network construction, functional enrichment analysis, and experimental validation studies.

(4) Functional enrichment analysis: The core intersecting gene set was subjected to Gene Ontology (GO) functional annotation and Kyoto Encyclopedia of Genes and Genomes (KEGG) pathway enrichment analyses using Metascape (https://metascape.org/), a high-throughput meta-database that consolidates functional annotation information from 40 independent knowledge bases for systematic biological term enrichment and pathway analysis.

### 2.2. Ethics Approval and Consent to Participate

This study is a secondary analysis of publicly available transcriptomic data retrieved from the Gene Expression Omnibus (GEO) database (accession numbers: GSE190452 and GSE256068). No new human subjects were recruited, and no new experiments involving humans or animals were conducted by the authors. Ethical approval and informed consent for the original data collection were obtained by the respective primary investigators of each dataset, as documented in their original publications and GEO submissions. Our use of these de-identified data complies with the GEO data usage policies and relevant ethical guidelines.

### 2.3. Transcriptomic Data Acquisition and Bioinformatic Analysis

Publicly available transcriptomic datasets were retrieved from the Gene Expression Omnibus (GEO) database to investigate the molecular signatures associated with seizure disorders based on our previous studies [12,13]. The primary discovery cohort, GSE256068, comprised RNA-sequencing (RNA-seq) profiles from human brain tissues, including 23 control samples and 121 samples from patients with seizures. For external validation of the identified diagnostic markers, the GSE143272 dataset was utilized, which contains gene expression profiles from peripheral blood samples of 51 healthy individuals and 91 patients with seizures. Raw data were subjected to log2 transformation and quantile normalization to ensure inter-sample comparability. To identify the molecular perturbations in seizure-affected tissues, differential expression analysis was performed between the control and seizure groups in the discovery cohort. Differentially expressed genes (DEGs) were identified using the R package “limma (version 3.34.9)”, with significance criteria set at an *p*-value < 0.05 and|log_2_(fold change)| ≥ 1. To elucidate the biological implications of these DEGs, Gene Ontology (GO) and Kyoto Encyclopedia of Genes and Genomes (KEGG) enrichment analyses were conducted to pinpoint significantly altered biological processes and signaling pathways.

### 2.4. Construction and Validation of a Diagnostic Machine Learning Signature

To develop a robust diagnostic model for seizure identification, an integrated machine learning (ML) pipeline was implemented based on our previous study [13]. We evaluated a total of 113 algorithm combinations by integrating various feature selection and classification methods, including but not limited to Random Forest (RF), Support Vector Machine (SVM), XGBoost, LASSO, Ridge, Elastic Net (Enet), and NaiveBayes based our previous studies. The GSE256068 dataset (brain tissue) served as the primary cohort and was randomly partitioned into a training set (70%) and a test set (30%). To ensure the generalizability of the models, the GSE143272 dataset (peripheral blood) was utilized as an independent external validation cohort. The diagnostic performance of each of the 113 combinations was quantified using the Area Under the Receiver Operating Characteristic (ROC) Curve (AUC). The mean AUC across the training, testing, and external validation cohorts was calculated to rank the stability and predictive power of the algorithm combinations. The combination with the highest and most consistent AUC was selected as the final diagnostic framework. Finally, the overlapping genes consistently identified by the top-performing algorithms were established as the core diagnostic signature.

### 2.5. Chemicals and Reagents

6PPDQ (HY-153169) was procured from MedChemExpress (MCE, Shanghai, China). A stock solution was prepared by dissolving 5 mg of 6PPDQ in dimethyl sulfoxide (DMSO). This stock was subsequently diluted in culture medium to achieve final working concentrations of 10 µg/L and 5 µg/L for cellular experiments. The final concentration of DMSO in all treatment groups was maintained below 0.1% (*v*/*v*), which is generally considered non-toxic and does not significantly alter these cellular endpoints. The primary antibody against IL-6 (DF6087) was obtained from Affinity Biosciences (Shanghai, China), whereas those targeting NFE2L2/Nrf2 (66504-1-Ig), TP53 (34129-1-AP), and β-actin (66009-1-Ig) were sourced from Proteintech (Shanghai, China).

### 2.6. Cell Lines and Culture Conditions

The murine hippocampal neuronal cell line HT22 (Catalog No. CL-0697) and the murine microglial cell line BV2 (Catalog No. CL-0493) were purchased from Procell Life Science & Technology Co., Ltd. (Wuhan, China) based on previous study [14]. Both cell lines were maintained in high-glucose Dulbecco’s Modified Eagle Medium (DMEM) (Gibco, Thermo Fisher Scientific, Waltham, MA, USA). Authentication of these cell lines was by short tandem repeat (STR) profiling and all experiments were conducted using cells between passages 5 and 10 to ensure consistent phenotypic and functional properties. The culture medium was supplemented with 10% heat-inactivated fetal bovine serum, 100 U/mL penicillin, and 100 μg/mL streptomycin. Cells were cultured in a humidified atmosphere containing 5% CO_2_ at 37 °C with relative humidity.

### 2.7. In Vitro Experimental Models and Pharmacological Interventions

To delineate the molecular pathways driving 6PPDQ-mediated neurotoxicity and its exacerbation of seizure-associated damage, we established two distinct in vitro experimental paradigms:

Direct Exposure Model: In this study, we used the immortalized HT22 hippocampal neuronal cell line to explore the effects of 6PPDQ on neuronal injury. A concentration of 5 mM glutamate was used to induce oxidative toxicity. This model mimics the severe oxidative stress and GSH depletion observed in vulnerable brain regions such as the hippocampus during seizure activity [15,16]. To assess the direct impact of 6PPDQ on neuronal vulnerability, cells were assigned to the following three cohorts: 1. Control group: Cells maintained in standard growth medium. 2. Glutamate (Glu) group: Cells exposed to 5 mM glutamate. 3. 6PPDQ + Glu group: Cells co-treated with 10 µg/L 6PPDQ and 5 mM glutamate to evaluate the synergistic potentiation of excitotoxic injury [10,17].

Indirect Exposure Model: Microglia–Neuron Crosstalk: To further investigate the role of neuroinflammation in 6PPDQ-induced damage, a microglia–neuron crosstalk model utilizing BV2 microglia and HT22 neurons was established. BV2 cells were stimulated with 5 µg/L or 10 µg/L 6PPDQ for 24 h to elicit a pro-inflammatory phenotype. Following incubation, the culture supernatants were collected and centrifuged to remove cellular debris. The resulting fluid was termed “6PPDQ-conditioned medium (BV2-CM)”. HT22 cells were challenged with 5 mM glutamate and segregated into four cohorts to evaluate the impact of microglial-mediated neurotoxicity: 1. Control group: HT22 cells maintained in standard vehicle medium. 2. Glutamate group: Cells exposed to 5 mM glutamate in fresh medium. 3. 5 µg/L 6PPDQ (BV2-CM) + Glu group: Cells co-incubated with 5 mM glutamate suspended in the 5 µg/L 6PPDQ-prepared BV2 CM. 4. 10 µg/L 6PPDQ (BV2-CM) + Glu group: Cells co-incubated with 5 µM glutamate suspended in the 10 µg/L 6PPDQ-prepared BV2 CM.

### 2.8. Pharmacological Rescue Experiments

To validate the mechanistic involvement of specific pathways, pharmacological interventions were applied. HT22 cells were pre-treated for 2 h with either the TP53 inhibitor Pifithrin-α (10 μm) or the antioxidant N-acetyl-L-cysteine (NAC, 5 mM) before exposure to 6PPDQ and glutamate [18,19].

### 2.9. Cell Viability Analysis

To evaluate half-maximal inhibitory concentration (IC_50_) of 6PPDQ-mediated cytotoxicity in a glutamate-induced excitotoxicity model, the Cell Counting Kit-8 (CCK-8) assay was performed in accordance with the standard procedures described previously [20]. Post-treatment, cell cultures were incubated with CCK-8 solution for 2 h at 37 °C. Optical density readings were subsequently acquired at 450 nm via a microplate reader, with data normalized to the glutamate-treated control group to calculate relative percentage viability.

### 2.10. Intracellular Glutathione Measurement

Intracellular reduced glutathione (GSH) levels were quantified using a GSH Assay Kit (S0053, Beyotime, Shanghai, China) according to the manufacturer’s instructions [12,21]. Briefly, following experimental treatments, HT22 cells were collected and lysed to obtain the supernatant. The concentration of GSH was determined by measuring the absorbance at 412 nm and results were expressed as a relative percentage of the control group.

### 2.11. Enzyme-Linked Immunosorbent Assay (ELISA)

To characterize the 6PPDQ-induced neuroinflammatory response, the release of the pro-inflammatory cytokine IL-6 from microglia was quantified based on our previous studies [11,12,20]. BV2 cells were exposed to 10 µg/L 6PPDQ for 24 h, after which the culture supernatants were collected and centrifuged to remove cellular debris. The IL-6 concentration was measured using a murine IL-6 ELISA kit (ml300479, Shanghai Enzyme-linked Biotechnology Co., Ltd., Shanghai, China) in strict accordance with the manufacturer’s instructions. Absorbance was recorded at 450 nm, and cytokine levels were determined based on a concurrently generated standard curve.

### 2.12. Measurement of Intracellular ROS Levels

Intracellular reactive oxygen species (ROS) levels in HT22 neurons were measured using the fluorescent probe DCFH-DA (Reactive Oxygen Species Assay Kit, Beyotime Biotechnology, Shanghai, China) [20,21]. Following designated treatments, cells were incubated with 10 μM DCFH-DA at 37 °C for 20 min in the dark, washed three times with serum-free medium, and immediately analyzed by flow cytometry on a BD FACSCanto™ II system (BD Biosciences, Franklin Lakes, NJ, USA,). For each sample, 10,000 viable single-cell events were acquired. The gating strategy included: (1) FSC-A vs. SSC-A to exclude cellular debris; (2) FSC-H vs. FSC-A to exclude cell doublets; and (3) FL1 channel (530/30 nm) to detect DCF fluorescence intensity. The following controls were included in each experiment: Negative control: Untreated cells without DCFH-DA staining (to assess autofluorescence); Positive control: Cells treated with 50 μg/mL Rosup (a ROS inducer provided in the kit) for 20 min prior to DCFH-DA loading. Fluorescence data were analyzed using FlowJo software (v10). The mean fluorescence intensity (MFI) of the FL1 channel was used to quantify ROS levels. MFI values from all treatment groups were normalized to the untreated control group (set as 1.0) within each independent experiment. All experiments were performed with five biological replicates (*n* = 5).

### 2.13. Immunofluorescence Staining

To visualize the spatial expression patterns of Nrf2, a pivotal transcription factor governing antioxidant defense mechanisms and neuroinflammatory pathways, immunofluorescence assays were carried out. The specific staining procedures were conducted in alignment with the experimental protocols detailed in our prior investigations [10,20].

### 2.14. Cellular Thermal Shift Assay (CETSA)

The physical interaction between 6PPDQ and the TP53 protein was evaluated using the CETSA method. HT22 cells were treated with 10 µg/L 6PPDQ or DMSO for 2 h, harvested, and distributed into PCR tubes [13,22]. The cell suspensions were heated across a temperature gradient (40–60 °C) for 3 min, followed by a 3 min cooling period at room temperature. After three freeze–thaw cycles in liquid nitrogen to achieve lysis, the soluble protein fractions were isolated via centrifugation. The presence of non-denatured TP53 in the supernatants was quantified by Western blot. Thermal stability was assessed by comparing the melting curves of the 6PPDQ-treated and control groups.

### 2.15. Surface Plasmon Resonance (SPR) Analysis

All SPR experiments were conducted at 25 °C using a Biacore 8K instrument (Cytiva, Marlborough, MA, USA) based on previous study. Recombinant TP53 protein was covalently immobilized onto CM5 sensor chips via amine coupling chemistry, achieving a surface density of approximately 2000 response units (RU). Serially diluted 6PPDQ (ranging from 0.3125 to 10 μM) was injected over both the active and reference flow cells at a flow rate of 30 μL/min in running buffer. The association and dissociation phases were monitored for 60 s and 140 s, respectively. Following double referencing to correct for bulk refractive index changes and non-specific binding, the sensograms were fitted to a 1:1 Langmuir binding model using BIAevaluation software Version 3.0. This analysis was performed to determine the equilibrium dissociation constant (KD).

### 2.16. Statistical Analysis

Quantitative data are presented as mean ± standard deviation (SD). The normality of data distribution was verified utilizing the Shapiro–Wilk test to determine appropriate statistical approaches. For datasets exhibiting a normal distribution, comparisons were conducted via unpaired *t*-tests or one-/two-way analysis of variance (ANOVA) followed by Tukey’s post hoc multiple comparison test. In contrast, non-parametric datasets necessitated analysis using the Mann–Whitney U test or Kruskal–Wallis test with Dunn’s correction for multiple comparisons. All statistical computations were undertaken using GraphPad Prism software (version 9.1), with statistical significance defined at an alpha level of *p* < 0.05. To ensure transparency and reproducibility, the full source code for analysis and visualization has been deposited in repository hosted on GitHub (https://github.com/PediatricLab-Jiangnan/6PPDQ-network, accessed on 1 May 2026).

## 3. Results

### 3.1. Identification of Shared Targets Linking 6PPDQ Exposure to the Exacerbation of Epilepsy-Induced Neuronal Damage

**Figure 1:**
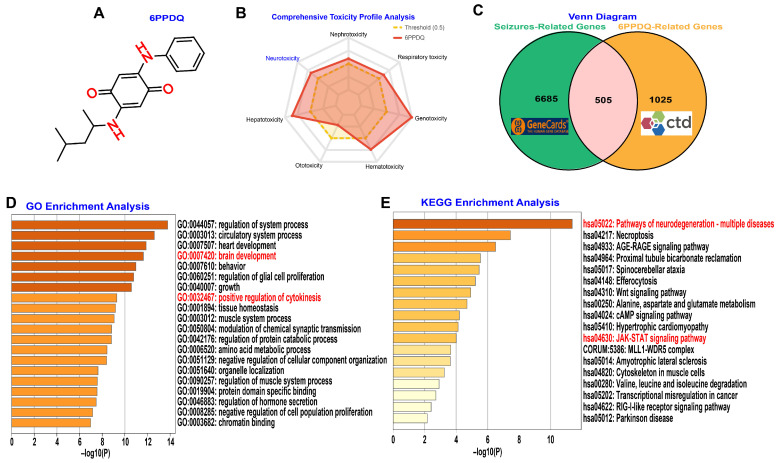
Comprehensive toxicological and functional characterization of 6PPD-quinone (6PPDQ). (**A**) Chemical structure of 6PPDQ. (**B**) Radar plot illustrating the comprehensive toxicity profile of 6PPDQ across multiple organ systems. (**C**) Venn diagram showing the overlapping genes. (**D**) GO enrichment analysis of the overlapping genes. (**E**) KEGG pathway enrichment analysis of the same gene set.

In this study, we initially conducted a comprehensive toxicological evaluation of 6PPDQ using the Admetlab 3.0 computational platform. The toxicity profile encompassed multiple organ systems, including neurotoxicity, nephrotoxicity, respiratory toxicity, genotoxicity, hematotoxicity, ototoxicity, and hepatotoxicity. Notably, this assessment revealed the potential neurotoxicity of 6PPDQ (Figure 1A,B). To elucidate the molecular mechanisms linking 6PPDQ exposure to seizure pathophysiology, we performed an integrative gene intersection analysis. By cross-referencing seizure-associated genes with 6PPDQ-interacting targets, we identified 505 overlapping genes, indicating substantial molecular convergence between 6PPDQ-induced toxicity and seizure disorders (Figure 1C). Subsequent functional enrichment analysis using Metascape revealed significant enrichment of GO biological processes related to “brain development” and “positive regulation of cytokinesis” (Figure 1D). These findings suggest that 6PPDQ may exacerbate seizure susceptibility by disrupting neurodevelopmental trajectories and modulating cytokinesis-related machinery. Furthermore, KEGG pathway analysis prioritized several critical signaling cascades, most notably the “JAK–STAT signaling pathway” and “Pathways of neurodegeneration” (Figure 1E). Given that the JAK–STAT axis serves as a canonical downstream effector of Interleukin-6 (IL-6) signaling, these data highlight the potential pivotal role of IL-6-mediated neuroinflammation in the etiology of 6PPDQ-associated neuropathology [23]. Collectively, these integrative computational analyses suggest that 6PPDQ may exert neurotoxic effects and promote seizure susceptibility primarily through inflammatory responses and neurodegenerative pathways.

**Figure 2:**
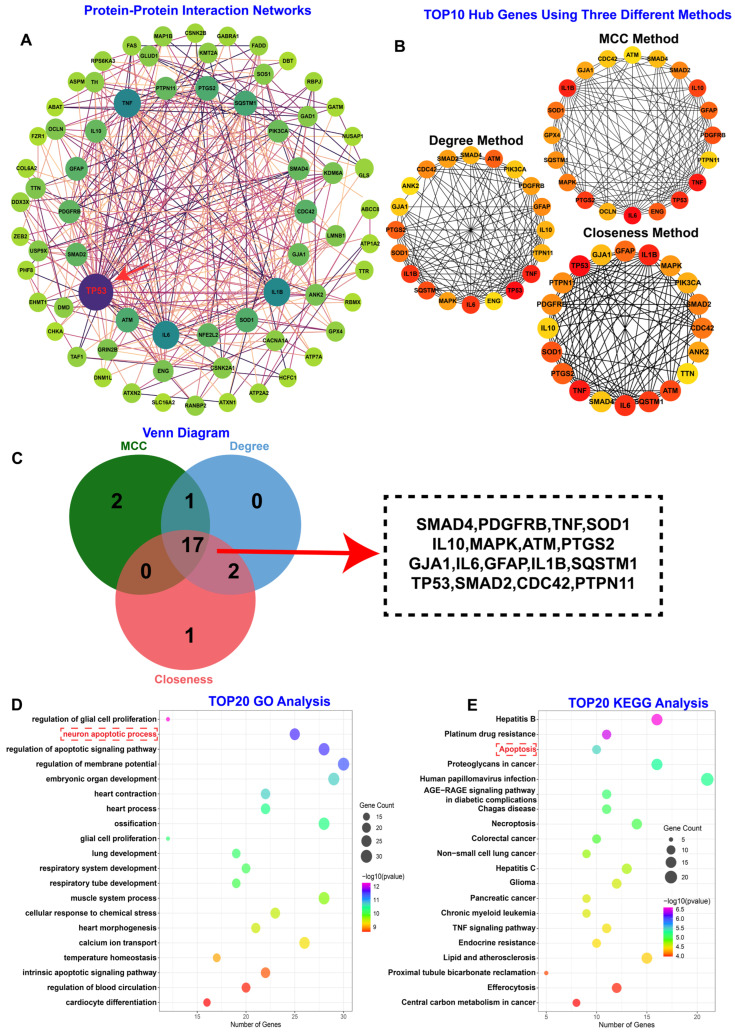
Identification and functional enrichment analysis of hub genes associated with 6PPDQ-induced neurotoxicity. (**A**) Protein–protein interaction network: The network illustrates the interaction landscape of differentially expressed genes. The node size and color intensity (from green to purple) are proportional to their connectivity degree, with TP53 identified as a prominent central node, suggesting its pivotal role in the regulatory network. (**B**) Topological analysis of hub genes: Sub-networks showing the top 20 candidate hub genes ranked by three different algorithms: MCC (Maximal Clique Centrality), Degree, and Closeness. Node colors ranging from yellow to red indicate increasing significance within the network, with redder nodes representing higher bottleneck or centrality properties. (**C**) Venn diagram for core target identification: The intersection of the top 20 genes from the three algorithms identified 17 common hub genes, ensuring the robustness of the hub gene selection. (**D**,**E**) Functional and Pathway Enrichment: GO (**D**) and KEGG (**E**) analyses of the 17 common hub genes. These results categorize the core targets into specific biological processes and molecular pathways, providing insights into the systematic impact of 6PPD-Q on the nervous system.

To discern the key molecular drivers within the 505 overlapping genes, we constructed a protein–protein interaction (PPI) network using the STRING database (Figure 2A). Notably, TP53 emerged as a central hub with the highest connectivity degree, suggesting its potential role as a dominant key regulator in 6PPDQ-associated seizure pathophysiology. To systematically prioritize core hub genes from this network, we applied three complementary topological algorithms using the CytoHubba plugin in Cytoscape (v3.10.2): MCC, Degree Centrality, and Closeness Centrality (Method S1). Each algorithm independently ranked the top 20 candidates (Figure 2B–D). The intersection of these three rankings yielded 17 consensus hub genes that were consistently prioritized across all methods, thereby minimizing algorithm-specific bias and enhancing confidence in target selection (Figure 2E). Subsequent GO and KEGG enrichment analysis of these 17 consensus hub genes revealed significant enrichment in biological processes related to “neuron apoptotic process” and “apotosis” (Figure 2E). These findings indicate that 6PPDQ primarily exerts its neurotoxic effects by triggering apoptotic pathways and impairing antioxidant defenses, the processes in which TP53 serves as a well-established master regulator of cellular stress and cell death. Collectively, these network topology analyses identify TP53 as a putative upstream regulator that links 6PPDQ exposure to increased neuronal vulnerability in epilepsy by coordinating apoptotic and oxidative stress responses.

### 3.2. 6PPDQ-Induced Neurotoxicity on Seizures Involving the Crosstalk Between Oxidative Damage and Neuroinflammation

**Figure 3:**
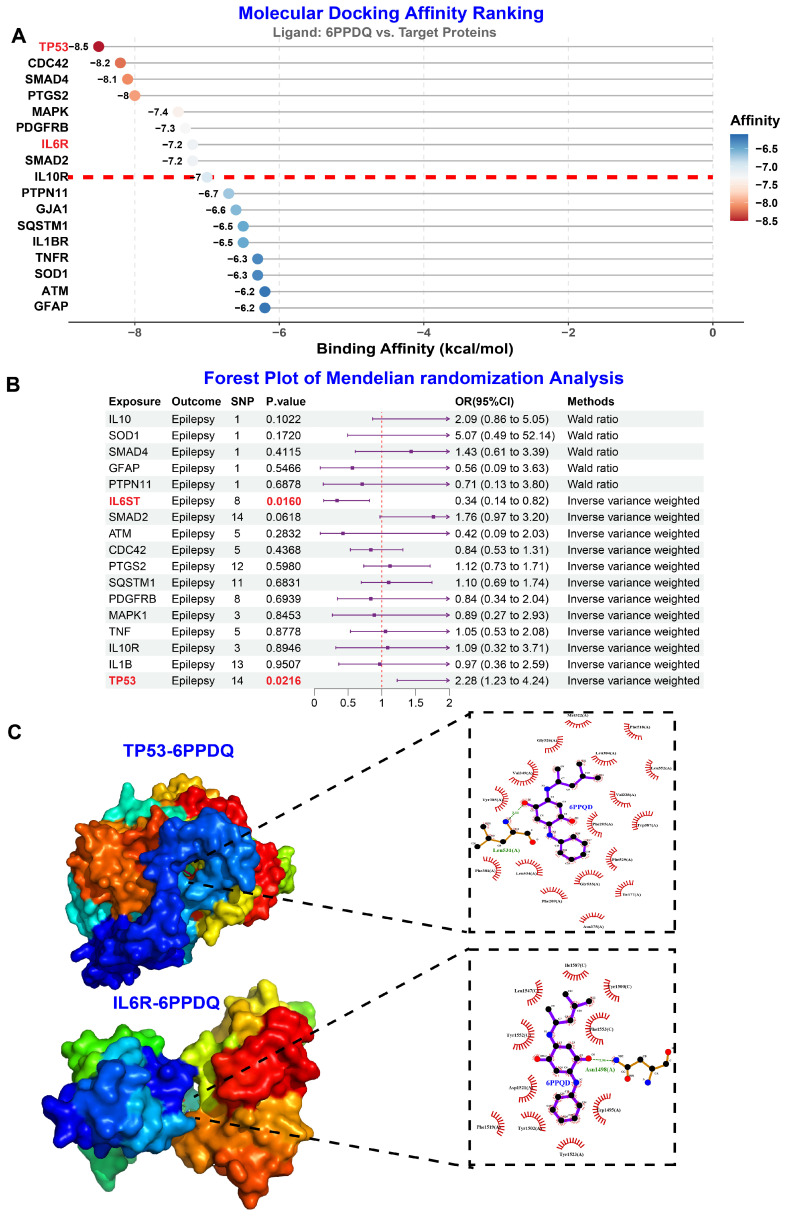
Validation of 6PPDQ–target interactions and causal association with epilepsy. (**A**) Molecular docking affinity ranking. The binding affinity (kcal/mol) of 6PPDQ with the 17 core hub proteins. The red dashed line denotes a high-affinity threshold. (**B**) Forest plot of two-sample Mendelian randomization analyses assessing causal effects of genetically predicted protein levels on epilepsy risk. Significant associations observed for IL6ST and TP53, supporting their roles as mediators of 6PPDQ-induced epileptogenesis. (**C**) Molecular docking visualization. Detailed 3D surface models and 2D ligand–residue interaction diagrams for the TP53-6PPDQ and IL6R-6PPDQ complexes.

To validate the direct molecular interactions between 6PPDQ and the core hub targets identified in our network analysis, we employed an integrative computational validation strategy combining molecular docking, Mendelian randomization (MR) analysis, and molecular dynamics (MD) simulations. We first performed molecular docking to evaluate the binding affinity between 6PPDQ and the 17 consensus hub genes (Method S2). A binding affinity threshold of −7.0 kcal/mol was established, as values below this cutoff are generally indicative of stable and biologically significant interactions [24]. Among these, TP53 and IL6R displayed the strongest binding energies and were prioritized for downstream validation (Figure 3A). To establish causal relationships between hub gene expression and seizure susceptibility, we performed two-sample Mendelian randomization analysis using GWAS summary statistics, and our findings provide genetic evidence supporting the causal role of TP53 and IL-6 signaling pathways in seizure pathophysiology (Method S3 and Figure 3B). Visual inspection of the docking poses confirmed the formation of stable hydrogen bonds and hydrophobic interactions within the active pockets of these proteins, thereby providing structural evidence for the direct engagement between 6PPDQ and its targets (Figure 3C).

**Figure 4:**
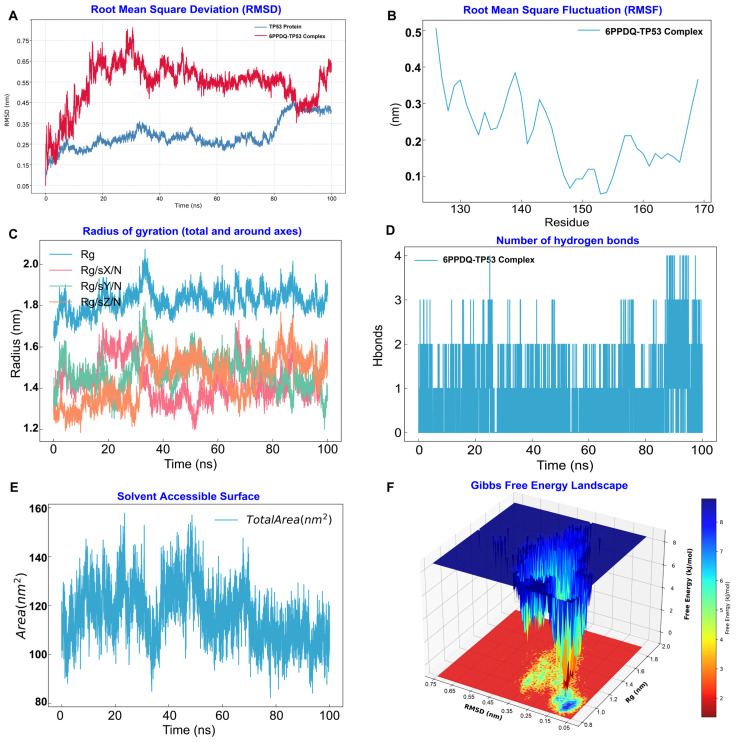
Molecular dynamics (MD) simulations characterizing the structural stability and conformational dynamics of the 6PPD-Q–TP53 complex. (**A**) Root Mean Square Deviation (RMSD): The RMSD profiles of the TP53 protein (blue) and the 6PPD-Q–TP53 complex (red) over a 100 ns trajectory. The plateauing of the curves suggests that the systems reached structural equilibrium during the simulation. (**B**) Root Mean Square Fluctuation (RMSF): Residue-specific fluctuations highlight the local flexibility of the TP53 protein. Low RMSF values in the binding region indicate reduced flexibility upon 6PPD-Q interaction. (**C**) Radius of Gyration (Rg): The total Rg (blue) and its components along the *x*, *y*, and *z* axes illustrate the structural compactness. Stable Rg values suggest that the complex remains densely packed without significant unfolding. (**D**) Intermolecular Hydrogen Bonds: The temporal evolution of the hydrogen bond count reflects the steady non-covalent interactions contributing to the binding affinity between 6PPD-Q and TP53. (**E**) Solvent Accessible Surface Area (SASA): The total area plot monitors the exposure of the complex to the solvent, providing insights into the hydrophobic core stability. (**F**) Gibbs Free Energy Landscape (FEL): A 3D energy surface projected onto the RMSD and Rg coordinates. The deep blue basins represent the most stable conformational states sampled during the simulation.

To further evaluate the thermodynamic stability and structural integrity of the top-ranked 6PPDQ-protein complexes under simulated physiological conditions, we performed 100 ns all-atom MD simulations (Method S4). The 6PPDQ-TP53 complex exhibited high conformational stability, as evidenced by multiple structural parameters: the RMSD of the complex stabilized at approximately 0.45 nm after 40 ns of simulation, indicating the achievement of a robust conformational equilibrium (Figure 4A). Analysis of the RMSF revealed elevated fluctuations in residues 130–170, which corresponds to increased flexibility within the DNA-binding domain upon 6PPDQ interaction (Figure 4B) [25]. The Rg was consistently maintained at approximately 1.8 nm, suggesting that the global compactness of the TP53 protein was preserved throughout the entire simulation period (Figure 4C). The stability of the binding interaction was further corroborated by the persistent formation of 2–4 hydrogen bonds between 6PPDQ and the TP53 binding pocket, which facilitated stable intermolecular anchoring (Figure 4D). The SASA remained relatively stable throughout the 100 ns trajectory, with no significant expansion or contraction observed (Figure 4E). Additionally, the Gibbs Free Energy Landscape displayed a single, deep energy minimum, confirming that the 6PPDQ-TP53 complex exists in a thermodynamically favorable and stable conformation (Figure 4F) [26]. Collectively, these computational findings identify TP53 as a primary molecular target of 6PPDQ. The stable binding of 6PPDQ to TP53 is likely to trigger downstream pathological signaling cascades, which may mediate 6PPDQ-induced neurotoxicity and exacerbate seizure severity.

**Figure 5:**
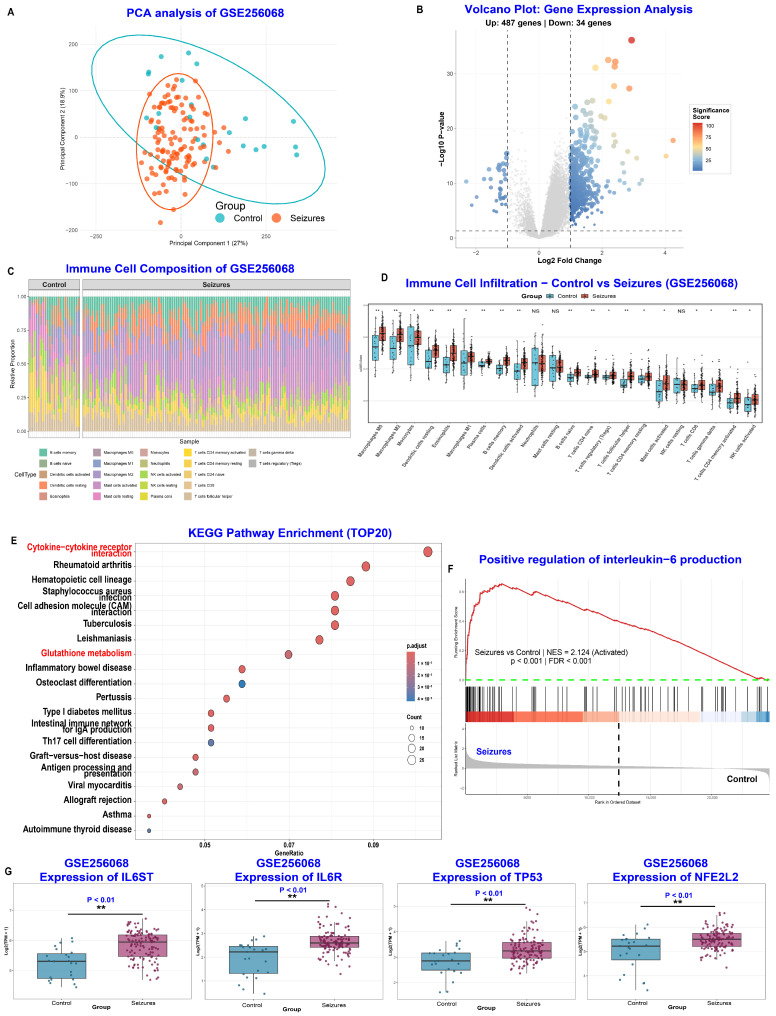
Transcriptomic landscape and immune infiltration analysis of the seizure model (GSE256068). (**A**) Principal Component Analysis (PCA) between the Control group and the Seizures group, indicating significant global transcriptomic shifts in the seizure model. (**B**) Volcano plot of differentially expressed genes (DEGs). (**C**,**D**) Immune cell infiltration analysis. (**C**) Stacked bar chart showing the relative proportion of 22 immune cell types across individual samples. (**D**) Box plot comparing the infiltration levels of specific immune cells between groups. (**E**) KEGG pathway enrichment of DEGs. (**F**) Gene Set Enrichment Analysis (GSEA). GSEA plot showing a significant enrichment of the “Positive regulation of interleukin-6 production” gene set in the Seizures group, suggesting that IL-6 mediated signaling is highly activated. (**G**) Expression levels of four core hub genes (IL6ST, IL6R, TP53, NFE2L2) across control vs. seizure groups. (NS not significant, * *p* < 0.05, ** *p* < 0.01).

As our prior findings identify TP53 as a central hub and the existing literature implicating Nrf2 (encoded by NFE2L2) as a critical regulator of oxidative stress and anti-inflammatory responses in epilepsy, we hypothesized that Nrf2 functions as a downstream effector modulated by TP53 in 6PPDQ-associated seizure pathophysiology [11,12,20]. To test this hypothesis and elucidate the underlying molecular landscape, we analyzed the GSE256068 dataset, comprising human brain tissue samples from 121 patients with seizures and 23 healthy controls. Principal Component Analysis revealed a pronounced spatial segregation between the seizure and control cohorts along the first two principal components, indicative of substantial global transcriptomic reprogramming associated with the disease state (Figure 5A). Volcano plot analysis identified a total of 531 DEGs, consisting of 497 upregulated and 34 downregulated genes in the seizure group (Figure 5B). Consistent with our postulated neuroinflammatory mechanism, immune cell infiltration analysis (Method S5) unveiled significant perturbations in the abundance of distinct immune cell populations. Specifically, we observed a marked increase in pro-inflammatory subsets, including activated microglia and M1 macrophages, alongside a reduction in neuroprotective populations (Figure 5C,D). Crucially, KEGG and GSEA prioritized “Glutathione metabolism” and “Positive regulation of interleukin-6 production” (NES = 2.124), reinforcing the link between oxidative stress and neuroinflammation (Figure 5E,F). Expression analysis confirmed that in the seizure-affected brain, TP53 is significantly upregulated while NFE2L2 (Nrf2) is relatively suppressed (Figure 5G). This reciprocal expression pattern supports our proposed mechanism: 6PPDQ exposure acts as a “second hit” that overactivates TP53, further dampening the Nrf2-mediated antioxidant defense. This synergy likely drives the excessive accumulation of ROS and exacerbates the neuronal damage associated with epileptic activity.

### 3.3. 6PPDQ Exposure Exacerbates Neuronal Injury Through a Synergistic Double-Hit Mechanism Involving the IL6/TP53/Nrf2 Pathway

**Figure 6:**
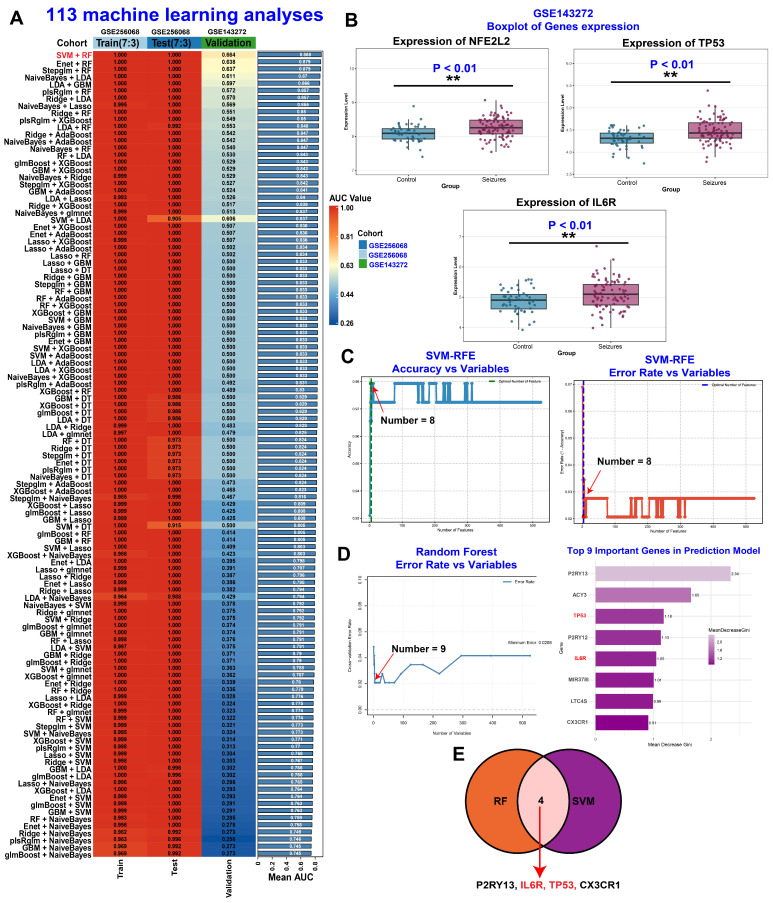
Development and validation of a diagnostic machine learning model for seizures. (**A**) Screening of 113 machine learning algorithm combinations. A heatmap and bar chart illustrating the performance (AUC values) of various combinations across training (GSE256068), testing, and external validation (GSE143272) cohorts. The SVM + RF (Support Vector Machine + Random Forest) combination exhibited the highest mean AUC and stability across all datasets. (**B**) Validation of hub gene expression in an external dataset (GSE143272). Box plots showing significantly higher mRNA expression levels of NFE2L2, TP53, and IL6R in the seizures group compared to the control group. (**C**) SVM-RFE analysis identifies optimal number of features for maximal classification accuracy. (**D**) RF analysis identifies optimal number of features for maximal classification accuracy. (**E**) Venn diagram illustrating overlap between top predictors selected by SVM-RFE and RF. ** *p* < 0.01.

To identify the most robust biomarkers associated with seizure pathophysiology, we applied a comprehensive machine learning framework building upon our previous methodologies [13,22]. We initially evaluated 12 distinct base algorithms and integrated their performance across 113 ensemble models. Among these, SVM and RF emerged as the top-performing approaches, demonstrating superior accuracy and generalizability (Figure 6A). Crucially, expression analysis in an independent external validation cohort (GSE143272, peripheral blood) corroborated the pivotal role of the TP53-Nrf2 axis in human seizure pathology, showing consistent dysregulation patterns observed in brain tissue (Figure 6B). Feature selection using both SVM-RFE (Figure 6C) and RF (Figure 6D) converged on four shared high-confidence genes: ACY3, IL6R, TP53, and CX3CR1 (Figure 6E). Notably, the consistent prioritization of IL6R reinforces our hypothesis that 6PPDQ exerts its neurotoxic effects, at least in part, by dysregulating IL-6 signaling. This dysregulation likely amplifies neuroinflammation and potentiates seizure pathology through the downstream TP53/Nrf2 pathway [27].

**Figure 7:**
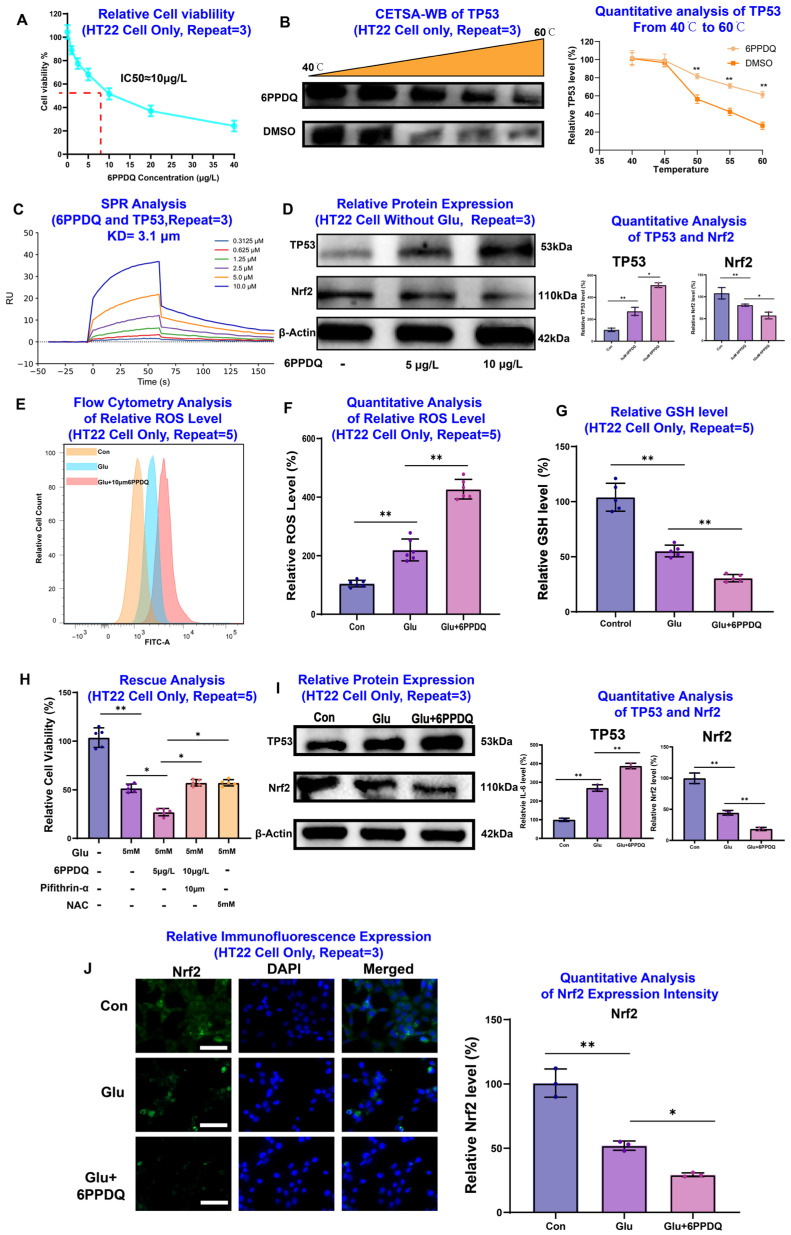
In vitro validation of 6PPDQ-induced neurotoxicity and the TP53/Nrf2 regulatory axis in HT22 cells. (**A**) Dose-dependent cytotoxicity: Relative cell viability of HT22 cells following 24 h exposure to varying concentrations of 6PPD-Q, with the IC50 determined for subsequent experiments. (**B**) Cellular Thermal Shift Assay (CETSA): Representative Western blot and corresponding thermal aggregation curves demonstrate the thermal stabilization of TP53 upon 6PPD-Q treatment, indicating the interaction between the ligand and the target protein. (**C**) Surface Plasmon Resonance (SPR) analysis: Sensorgrams illustrating the binding kinetics and affinity (KD value) of 6PPD-Q to immobilized TP53 protein. (**D**) Protein expression under baseline conditions: Western blot analysis showing the dose-dependent effects of 6PPD-Q on TP53 and Nrf2 protein levels in HT22 cells in the absence of glutamate. (**E**–**G**) Assessment of oxidative stress: Representative flow cytometry histograms (**E**) and quantitative analysis (**F**) of intracellular reactive oxygen species (ROS) levels, alongside the measurement of glutathione (GSH) content (**G**), revealing 6PPD-Q-exacerbated oxidative damage. (**H**) Rescue analysis: Cell viability assays utilizing specific inhibitors (Pifithrin-alpha) and antioxidants (NAC) to verify the involvement of the TP53/Nrf2 axis in 6PPD-Q-induced neurotoxicity. (**I**) Regulatory axis validation under oxidative stress: Western blot analysis demonstrating that 6PPD-Q significantly potentiates TP53 expression while further suppressing Nrf2 levels in glutamate-treated HT22 cells. (**J**) Immunofluorescence imaging: Representative imaging and quantification show a marked reduction in Nrf2 fluorescence intensity. Scale bar = 50 μm. Data are presented as mean ± SD; repeat times are indicated as *N* = 3 or *N* = 5 in the panels; * *p* < 0.05, ** *p* < 0.01.

**Figure 8:**
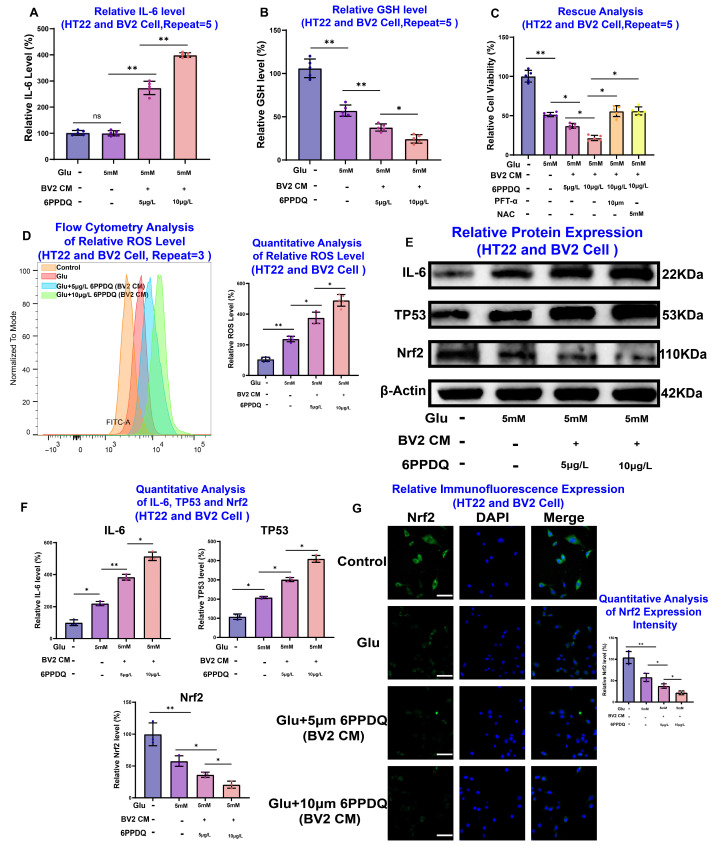
6PPDQ-activated BV2 microglia-derived inflammation exacerbates neurotoxicity in HT22 cells via the IL-6/TP53/Nrf2 axis. (**A**,**B**) Pro-inflammatory and oxidative markers. Treatment of HT22 cells with 6PPDQ-conditioned BV2 supernatant significantly increased (**A**) IL-6 levels and decreased (**B**) GSH levels in a dose-dependent manner compared to the glutamate (Glu) group. (**C**) Rescue analysis in the co-culture model. The reduction in HT22 cell viability induced by 6PPDQ-BV2 supernatant was significantly reversed by the TP53 inhibitor Pifithrin-α or the antioxidant NAC. (**D**) Quantitative analysis of intracellular reactive oxygen species (ROS) levels using DCFH-DA staining. (**E**,**F**) Western blot validation of the protein expression of IL-6, TP53 and Nrf2. (**G**) Immunofluorescence staining visualizes the expression intensity of Nrf2. Scale bar = 50 μm. (NS not significant, * *p* < 0.05, ** *p* < 0.01).

To experimentally validate these insights, we utilized a 5 mM glutamate-induced excitotoxicity model in HT22 hippocampal neurons, which is a gold-standard in vitro paradigm for studying epilepsy-related neuronal damage [21]. A CCK-8 assay showed that 6PPDQ induced dose-dependent toxicity with an IC50 of approximately 10 μM (Figure 7A). Notably, we characterized the direct interaction between 6PPDQ and TP53 using CETSA-WB and SPR. The results demonstrated that 6PPDQ significantly stabilized the TP53 protein against thermal denaturation (Figure 7B). Furthermore, SPR analysis revealed a high-affinity binding interaction with an equilibrium dissociation constant (KD) of 3.1 μM (Figure 7C). These results provide potential biophysical evidence of direct ligand–target engagement. Western blot analysis also demonstrated that 6PPDQ exposure alone triggered a dose-dependent increase in TP53 and a decrease in Nrf2 even without glutamate treatment, confirming that 6PPDQ possesses inherent neurotoxic potential that primes neurons for further damage (Figure 7D). Under excitotoxic stress, 6PPDQ significantly amplified glutamate-induced ROS accumulation (Figure 7E,F) and depleted intracellular GSH (Figure 7G). This effect was functionally linked to the TP53/Nrf2 axis as pharmacological inhibition of TP53 via Pifithrin-α or ROS scavenging via NAC successfully rescued HT22 cells from 6PPDQ-augmented death (Figure 7H). Western blot and immunofluorescence further confirmed that 6PPDQ exacerbates the reciprocal dysregulation of TP53 upregulation and Nrf2 suppression (Figure 7I,J). Finally, we established a microglia–neuron crosstalk model using BV2-conditioned medium (BV2-CM) to investigate the indirect inflammatory contribution. 6PPDQ stimulation induced a robust, dose-dependent increase in IL-6 secretion from BV2 cells (Figure 8A). When HT22 neurons were exposed to this 6PPDQ-stimulated BV2-CM, they exhibited severe oxidative stress, GSH depletion, and reduced viability (Figure 8B–D). These neurotoxic effects were successfully reversed by Pifithrin-α or NAC, confirming that microglial-derived inflammatory mediators converge on the neuronal TP53 pathway (Figure 8E–G). In summary, our findings elucidate a double-hit mechanism where 6PPDQ directly engages neuronal TP53 while simultaneously triggering microglial IL-6 release. These two pathways synergistically suppress Nrf2-mediated antioxidant defenses, ultimately leading to catastrophic neuronal damage and providing a mechanistic explanation for how tire-derived contaminants exacerbate seizure-related pathology.

## 4. Discussion

For over two decades, the mass mortality of returning Coho salmon in the Pacific Northwest remained an ecological enigma, characterized by mortality rates of up to 90% within hours of entering urban streams. Affected fish displayed acute neurological distress, distinctively marked by distinctive behavioral symptoms including surface swimming, loss of orientation, gaping (mouth opening), and loss of equilibrium before death [28,29]. The definitive identification of 6PPD-quinone (6PPDQ), a transformation product of the tire antioxidant 6PPD, as the causative agent has successfully elucidated this long-standing mystery [3]. While 6PPDQ is now recognized as a potent environmental toxicant driving acute aquatic mortality, emerging evidence indicates that its toxicity extends beyond aquatic species to disrupt fundamental cellular processes in mammals, raising significant concerns regarding its neurotoxic potential in humans. Crucially, a 2024 study analyzing CSF samples from Parkinson’s disease patients found that 6PPDQ levels were twice as high in PD patients compared to controls, and the compound was associated with abnormal α-synuclein aggregation in dopaminergic neurons [30]. Despite the fact that children are particularly vulnerable due to frequent exposure sources, such as recycled tire crumb rubber in playgrounds, and the developmental immaturity of their BBB, the specific contribution of 6PPDQ to pediatric seizure pathology remains poorly understood. To bridge this critical knowledge gap, the present study integrates network toxicology and machine learning-driven biomarker discovery with experimental validation. By systematically elucidating the mechanisms of 6PPDQ-induced damage, we focus on the synergistic interplay between neuroinflammation and oxidative stress mediated by the TP53/Nrf2 axis.

Neuroinflammation is a well-established hallmark of epileptogenesis, where the excessive release of pro-inflammatory cytokines lowers the seizure threshold and perpetuates neuronal injury [31]. Recent evidence indicates that 6PPDQ directly compromises the integrity of the blood–brain barrier (BBB), which serves as a critical interface restricting the entry of circulating immune cells and toxins into the central nervous system [32]. Upon BBB disruption by 6PPDQ, resident immune cells such as microglia detect the breach and mount a robust inflammatory response, releasing key cytokines including TNF-alpha, IL-1 beta, and IL-6. This sustained neuroinflammatory state is a recognized contributor to neurodegeneration and is closely linked to the pathophysiology of chronic seizures [7,32]. Furthermore, 6PPDQ-induced BBB breakdown permits the extravasation of serum albumin and potassium into the brain parenchyma. The uptake of albumin by astrocytes triggers transforming growth factor-beta signaling, which leads to the downregulation of potassium channels and impaired potassium buffering [33]. Concurrently, elevated extracellular potassium depolarizes neurons and fosters a state of local hyperexcitability, representing a key mechanism underlying the development of acquired epilepsy [32,34]. We therefore hypothesize that when 6PPDQ exposure occurs in individuals with pre-existing epileptic conditions, the convergent elevation of IL-6 from both seizure activity and environmental pollutants creates a synergistic neuroinflammatory state. This synergy likely amplifies neuronal damage through the pathogenic crosstalk between neuroinflammation and oxidative stress. Our study specifically identifies this process as a central driver of 6PPDQ-mediated neurotoxicity, where the IL-6-driven inflammatory environment further exacerbates the suppression of the Nrf2 antioxidant defense system.

Oxidative stress is a critical driver of seizure-induced neuronal damage. Status epilepticus leads to the persistent overproduction of reactive oxygen species, which contributes directly to neuronal death [35]. When 6PPDQ acts as an environmental pro-oxidant, the resulting synergistic oxidative burden can overwhelm the brain’s limited antioxidant capacity, amplifying neuronal injury beyond the levels caused by either factor alone. In this study, our integrative network toxicology analysis revealed a substantial molecular convergence between 6PPDQ-associated targets and genes implicated in epilepsy. Notably, KEGG enrichment highlighted the JAK-STAT signaling pathway as a central mediator, while PPI network analysis identified TP53 as a dominant hub regulator orchestrating the interplay between inflammatory responses and redox homeostasis. The IL-6/JAK-STAT axis is a pivotal driver of epileptogenesis. IL-6 is rapidly upregulated during seizure activity and contributes to neuronal hyperexcitability by modulating synaptic transmission and ion channel function [36]. IL-6 is rapidly upregulated during seizure activity and contributes to neuronal hyperexcitability by modulating synaptic transmission and ion channel function. Clinical evidence underscores this link, as serum IL-6 levels rise significantly within 3 to 24 h following a seizure, with the magnitude of elevation correlating with both seizure severity and duration [37]. Furthermore, persistently elevated interictal levels of IL-6 in patients with temporal lobe epilepsy are associated with higher seizure frequency and greater disease burden [38]. Our findings suggest a novel mechanistic link wherein environmental exposure to 6PPDQ primes this pro-inflammatory cascade.

Seizures are characterized by massive ATP release and excitotoxic stress, both of which can activate TP53 in neurons [39]. We propose that 6PPDQ-triggered TP53 activation may directly fuel the post-seizure cytokine storm, including the overexpression of IL-6. This excessive neuroinflammation not only contributes to blood–brain barrier disruption but also drives secondary damage by lowering the threshold for subsequent seizure episodes. Central to this mechanism is the antagonistic relationship between TP53 and Nrf2 [40]. Under physiological conditions, Nrf2 serves as the master regulator of antioxidant defense to maintain cellular redox balance. However, under conditions of severe toxicant exposure, hyperactivated TP53 can suppress Nrf2 transcriptional activity and promote its degradation [40]. Our data support the hypothesis that 6PPDQ exposure initiates a vicious cycle. Specifically, 6PPDQ directly stabilizes TP53 and upregulates IL-6, while elevated TP53 levels simultaneously suppress the Nrf2-mediated antioxidant axis. This leads to a critical failure in endogenous defense mechanisms, where the resulting accumulation of ROS further activates neuroinflammatory pathways [41]. Our data support the hypothesis that 6PPDQ exposure initiates a vicious cycle. Specifically, 6PPDQ directly stabilizes TP53 and upregulates IL-6, while elevated TP53 levels simultaneously suppress the Nrf2-mediated antioxidant axis. This leads to a critical failure in endogenous defense mechanisms, where the resulting accumulation of ROS further activates neuroinflammatory pathways.

To further elucidate the roles of IL-6, TP53, and Nrf2 in seizure pathophysiology, we performed the bulk RNA-seq analysis, our bulk transcriptomic data confirmed significant dysregulation of the IL-6/TP53/Nrf2 axis, with expression patterns strongly consistent with their proposed functions in neuroinflammation and oxidative stress pathways. Complementing these findings, our machine learning framework consistently prioritized IL-6 and TP53 as high-confidence biomarkers of seizure-associated neurotoxicity. Together, these multi-layered analyses support the hypothesis that 6PPDQ exposure exacerbates seizure pathology by amplifying neuroinflammation and disrupting redox homeostasis through the TP53/Nrf2 signaling axis [42]. Oxidative stress is inextricably linked to epileptogenesis as it acts as both a trigger and a consequence of neuronal hyperexcitability [43]. In this scenario, TP53 serves as a critical molecular hub that senses ROS surges and executes cell death programs. The conversion of oxidative stress into neuronal loss represents a key driver of epileptogenesis, establishing a feed-forward loop in which ROS accumulation exacerbates neuronal excitability, which in turn generates further ROS. Previous studies have demonstrated that status epilepticus induces severe oxidative stress and DNA damage in hippocampal neurons, leading to TP53 stabilization. Once activated, TP53 acts as the “molecular brake” on the Nrf2-mediated antioxidant system via two distinct mechanisms: first, TP53 induces p21 expression, which directly binds and sequesters Nrf2, thereby inhibiting its transcriptional activity, promoting ROS accumulation, and ultimately triggering cell death. Second, TP53 suppresses the expression of SLC7A11 [44]. The resulting depletion of intracellular cystine leads to glutathione exhaustion and sensitizes neurons to ferroptosis, which is an iron-dependent form of regulated cell death.

The clinical significance of this mechanism is underscored by observations in patients with temporal lobe epilepsy (TLE) and hippocampal sclerosis. Individuals with poor seizure control (ILAE Class 2) exhibit significantly reduced expression of Nrf2 and its downstream targets (HO-1, NQO1) compared to seizure-free individuals (ILAE Class 1) [45]. Critically, this deficit persists despite ongoing oxidative stress, as evidenced by elevated markers of lipid peroxidation and DNA oxidation. This suggests a maladaptive failure of the TP53/Nrf2 pathway rather than a mere insufficiency of antioxidant capacity. Such impairment leaves neurons defenseless against excitotoxic insults, promotes mitochondrial dysfunction, and facilitates the transition from acute seizures to chronic epilepsy. Our study also highlights the intricate crosstalk between inflammation and oxidative stress driven by 6PPDQ. The relationship between oxidative stress and neuroinflammation in epilepsy is not merely correlative but forms a self-reinforcing pathological feedback loop that exacerbates neuronal hyperexcitability and disease progression. Specifically, oxidative stress can trigger neuroinflammatory responses through the activation of the NF-κB signaling pathway, a master regulator of pro-inflammatory gene expression. Once activated, NF-κB translocates to the nucleus and promotes the transcription of cytokines such as IL-6, both of which are key mediators of neuroinflammation [46]. Conversely, pro-inflammatory cytokines can suppress the expression of cystine transporters, leading to GSH depletion in neurons. This bidirectional interplay creates a vicious cycle wherein oxidative stress fuels inflammation and neuroinflammation in turn exacerbates oxidative damage. This cycle eventually overwhelms endogenous defense systems, leading to lipid peroxidation and irreversible DNA damage. Collectively, these processes contribute to the initiation and propagation of seizures, ultimately driving the transition to chronic neurological dysfunction [47]. By positioning 6PPDQ as a potent trigger of this cycle, our findings provide a mechanistic framework for how environmental contaminants intensify the underlying pathology of epilepsy.

## 5. Limitation

Despite the significant mechanistic insights generated by this study, several limitations warrant acknowledgment to contextualize our findings and guide future research directions. First, although our microglia–neuron co-culture system effectively recapitulates key aspects of neuroimmune crosstalk and paracrine signaling, it lacks the three-dimensional architectural complexity of the intact blood–brain barrier, the diversity of non-neuronal cell types, and the functional synaptic connectivity present in the living brain. Consequently, our model may not fully capture systemic metabolic clearance, neurovascular unit interactions, or the network-level hyperexcitability that defines clinical seizures. Future studies should prioritize validating these findings in vivo using established rodent models of acute and chronic epilepsy combined with telemetry EEG monitoring. Such approaches will be critical to assess the systemic pharmacokinetics, circuit-level impact, and long-term epileptogenic potential of 6PPDQ exposure. Second, a notable limitation is the use of immortalized HT22 hippocampal neuronal cells and BV2 microglial cells. While convenient and reproducible, these cell lines do not fully recapitulate the complexity of the intact brain. Specifically, HT22 cells lack functional ionotropic glutamate receptors; therefore, the glutamate-induced injury model employed here reflects cell death driven by cystine uptake inhibition and GSH depletion rather than classical receptor-mediated excitotoxicity that occurs during seizures in vivo. Furthermore, although our experimental design utilized acute, high-dose exposure paradigms to effectively elucidate mechanistic interactions within the IL-6/TP53/Nrf2 axis, this approach deviates from real-world environmental conditions. Our concentrations were selected to probe molecular pathways and establish proof-of-concept regarding target engagement; however, they do not accurately reflect the low-dose, chronic exposure scenarios typically encountered in urban environments. Consequently, while our data robustly support the biological plausibility of 6PPDQ-induced neurotoxicity, direct extrapolation of these specific dosage metrics to human environmental risk assessment is limited. Future validation in primary neuron–glia co-cultures or in vivo seizure models, coupled with investigations into the long-term effects of environmentally relevant concentrations, is essential to confirm translational relevance and determine if sustained, low-level exposure results in cumulative molecular damage or functional deficits. Third, although our focus on the IL-6/TP53/Nrf2 axis was guided by convergent evidence from network toxicology, Mendelian randomization, and experimental validation, our integrative analysis suggests that 6PPDQ may interact with a broader spectrum of molecular targets. Potential off-target effects involving mitochondrial dynamics, calcium homeostasis, and synaptic vesicle cycling were hinted at in our KEGG enrichment but not explicitly dissected in this study. In addition, the current study did not specifically investigate Nrf2 isoforms or post-translational modifications. The differential Nrf2 banding patterns observed under distinct inflammatory and oxidative stress conditions may potentially reflect alterations in protein stability, degradation status, or post-translational processing. However, the precise molecular basis underlying these observations remains unclear and requires further mechanistic investigation. Future multi-omics studies integrating proteomics, phosphoproteomics, and metabolomics may help uncover additional synergistic pathways contributing to the full neurotoxic profile of 6PPDQ. Fourth, our study utilized standardized cell lines and public transcriptomic datasets, which do not fully account for inter-individual genetic variability. In real-world scenarios, factors such as genetic polymorphisms in detoxification enzymes, antioxidant genes, or inflammatory regulators may significantly modulate individual susceptibility to 6PPDQ-induced neurotoxicity. Future epidemiological studies incorporating gene–environment interaction analyses are needed to identify vulnerable subpopulations who may be at disproportionately higher risk. Finally, our experimental design focused on acute to sub-acute exposure windows. However, environmental exposure to tire wear particles is often chronic and occurs during critical windows of neurodevelopment (e.g., prenatal, early childhood). The long-term consequences of low-dose, chronic 6PPDQ exposure on neuronal maturation, synaptic pruning, and cognitive development remain unknown. Longitudinal studies spanning developmental stages are crucial to determine whether early-life exposure predisposes individuals to late-onset neurological disorders beyond epilepsy, such as neurodegenerative diseases or cognitive deficits. Notwithstanding these limitations, this study provides the first comprehensive evidence linking 6PPDQ to seizure pathophysiology via the IL-6/TP53/Nrf2 axis, offering a vital foundation for subsequent in vivo validation, biomonitoring efforts, and regulatory policy development.

## 6. Conclusions

In conclusion, this study integrates multi-omics computational predictions with rigorous in vitro experimental validation to unveil a novel mechanism by which 6PPDQ, an emerging tire wear-derived environmental contaminant, exacerbates seizure-associated neurotoxicity. Our findings demonstrate that 6PPDQ exerts its neurotoxic effects through a synergistic double-hit mechanism involving direct binding to and stabilization of neuronal TP53 alongside the indirect induction of microglial IL-6 secretion. These two converging pathways suppress the master antioxidant regulator Nrf2, leading to unchecked ROS accumulation, compromised glutathione metabolism, and ultimately, accelerated neuronal damage. By providing biophysical evidence of direct ligand–target engagement between 6PPDQ and TP53, this research establishes a robust mechanistic link between environmental chemical exposure and the aggravation of excitotoxic injury. Given the ubiquitous presence of 6PPDQ in tire wear particles and its confirmed detection in playgrounds and urban runoff, our results underscore a significant environmental risk factor for the pediatric population. Children remain particularly vulnerable to such contaminants due to the high plasticity and unique physiological characteristics of the developing brain. Furthermore, by identifying the TP53/Nrf2 axis as a critical vulnerability point, this work highlights promising therapeutic strategies such as pharmacological TP53 inhibition or Nrf2 activation to mitigate pollutant-mediated neurological damage. Ultimately, this research bridges the gap between environmental toxicology and clinical neurology. It provides crucial mechanistic evidence to inform public health policies, refine age-specific risk assessment frameworks, and drive regulatory actions aimed at reducing human exposure to tire-derived contaminants. As the global use of synthetic rubber continues to rise, understanding and mitigating the neurotoxic impacts of transformation products like 6PPDQ remains imperative for safeguarding neurological health in vulnerable populations.

## Data Availability

The raw data supporting the conclusions of this article will be made available by the authors on request.

## Supplementary Materials

The following supporting information can be downloaded at: https://www.mdpi.com/article/10.3390/toxics14050443/s1. References [9,10,11,13,20,22] are cited in the Supplementary Materials.
