# Supplementary material for: 6PPDQ Exposure Exacerbates Seizure-Induced Neuronal Damage via the TP53/Nrf2 Axis: An Integrated Strategy Combining Network Toxicology and Experimental Validation
Source: Toxics. 2026 May 19;14(5):443. doi: 10.3390/toxics14050443 (PMC13211372; doi:10.3390/toxics14050443)
Supplement: Supplementary file 1 [file toxics-14-00443-s001.zip › toxics-4297411-supplementary.pdf]

Supplemental Methods Section

1. Protein–Protein Interaction (PPI) Network Construction

To explore the functional interrelationships among the intersecting candidate genes, a protein–protein interaction (PPI) network was constructed using the STRING database (https://string-db.org/), a specialized resource for systematically mining and annotating known and predicted protein–protein associations. The constructed PPI network was subsequently visualized and subjected to topological analysis using Cytoscape (v3.10.2) with the CytoHubba plugin. Hub genes were screened based on three classic topological algorithms, namely Maximal Clique Centrality (MCC), Degree Centrality, and Closeness Centrality. Genes that were consistently ranked in the top tier across all three aforementioned algorithms were defined as core hub genes.

2. Molecular Docking and Visualization

To explore the potential binding modes and interaction characteristics between 6PPDQ and the identified hub proteins, molecular docking simulations were performed based on our previous study. The three-dimensional crystal structures of the target hub proteins were obtained from the RCSB Protein Data Bank (PDB). Molecular docking was carried out using AutoDock Vina (v1.1.2) with its built-in iterated local search global optimization algorithm. Binding affinities were quantified based on the lowest binding free energy (kcal/mol), where more negative values indicated stronger binding interactions between 6PPDQ and the target proteins. Docking conformations were ranked according to binding affinity, and poses with a binding affinity ≤ -7.0 kcal/mol were defined as high-confidence predictions, in accordance with commonly accepted

thresholds in structure-based drug design and computational toxicology.

3. Mendelian Randomization Analysis

To assess the potential causal relationship between the expression of the identified hub genes and seizure susceptibility, a two-sample Mendelian randomization (MR) analysis was conducted. Genetic instruments corresponding to the expression quantitative trait loci (eQTLs) of the hub genes were retrieved from the IEU OpenGWAS database (https://gwas.mrcieu.ac.uk/), a specialized repository providing comprehensive GWAS summary data for genetic association studies. Summary-level GWAS datasets for seizure disorders were obtained from the NHGRI-EBI GWAS Catalog (https://www.ebi.ac.uk/gwas/), which curates high-quality human genome-wide association study data with standardized genetic annotations. The Inverse Variance Weighted (IVW) method was employed as the primary analytical approach to estimate the causal effect of hub gene expression on seizure risk; for single-nucleotide polymorphism (SNP) instruments with a sample size of 1, the Wald ratio method was applied for supplementary causal inference. All MR analyses were implemented using the TwoSampleMR R package (R version 4.4.1), a dedicated tool for two-sample Mendelian randomization analyses with GWAS summary data. The results were reported as odds ratios (OR) with 95% confidence intervals (CI), and a P-value < 0.05 was defined as the threshold for statistical significance.

4. Molecular Dynamics Simulations

To assess the thermodynamic stability and conformational changes in the protein–6PPDQ complexes under physiological conditions, 100 ns MD simulations were

performed using GROMACS v2023.2 with the CHARMM36m force field based on our previous study. The stability and flexibility of the complex were evaluated using the following parameters: Root Mean Square Deviation (RMSD) and Root Mean Square Fluctuation (RMSF), Radius of Gyration (Rg) and Solvent Accessible Surface Area (SASA). Gibbs Free Energy Landscape (FEL) analysis was used to determine the lowest energy states.

5. Immune Microenvironment Analysis

The immune infiltration landscape of the seizure-affected brain was characterized using the CIBERSORT algorithm, which deconvolutes the relative proportions of 22 distinct immune cell types based on validated gene expression signatures. Furthermore, Gene Set Enrichment Analysis (GSEA) was performed to evaluate the activation status of specific functional pathways, such as interleukin-6 production and inflammatory responses, providing a more granular view of the disease-associated molecular shifts.

6. Quantitative real-time PCR(Rt-qPCR)

Total RNA was isolated from HT22 using TRIzol reagent (Invitrogen, USA) according to the manufacturer's instructions. RNA concentration and purity were assessed by spectrophotometry (A260/A280 ratio >1.9). Complementary DNA (cDNA) was synthesized from 1 μg of total RNA using a PrimeScript™ RT Master Mix (Takara, Japan) in a 20 μL reaction volume. Quantitative real-time PCR (RT-qPCR) was performed using SYBR Green Premix Pro Taq HS qPCR Kit (Accurate Biotechnology, China) on a CFX96 Real-Time PCR Detection System (Bio-Rad, USA). The primer sequences for PTGS2 (prostaglandin-endoperoxide synthase 2, also known as COX-2)

were as follows: Forward: 5′-TTCCAATCCATGTCAAAACCGT-3′ , 5′-AGTCCGGGTACAGTCACACTT-3′ (Reverse). Amplification was carried out under the following thermal cycling conditions: Initial denaturation at 95°C for 30 seconds, followed by 40 cycles of 95°C for 5 seconds and 60°C for 30 seconds. Melting curve analysis confirmed the presence of a single specific amplicon. GAPDH was used as the reference gene for normalization. Relative gene expression was calculated using the ΔΔCt method. All experiments were performed with three independent biological replicates, and each sample was run in triplicate technical replicates to ensure reproducibility.

7. Western blot analysis

Western blot analyses were performed to assess the expression levels of key proteins in HT22 cells following 6PPDQ exposure. Total protein was extracted using ice-cold RIPA lysis buffer (Beyotime, China) supplemented with protease and phosphatase inhibitor cocktails. Protein concentrations were quantified via BCA assay (Beyotime, China). Equal amounts of protein (20 μg per lane) were separated on 10% SDS-PAGE gels and transferred onto PVDF membranes. Membranes were blocked with 5% (w/v) non-fat milk in TBST for 1 h at room temperature and then incubated overnight at 4°C with primary antibodies diluted 1:1000 in blocking buffer: Anti-TP53 (Proteintech, 34129-1-AP) Anti-NFE2L2/Nrf2 (Proteintech, 66504-1-Ig), Anti-IL-6 (Affinity Biosciences, DF6087), Anti-β-actin (Proteintech, 66009-1-Ig; loading control). After washing, membranes were incubated for 1 h at room temperature with HRP-conjugated secondary antibodies (diluted 1:10000 in 5% milk/TBST). Protein bands were

visualized using an ECL chemiluminescence detection system (Beyotime, China). Densitometric quantification was performed using ImageJ software (NIH, USA). Band intensities of target proteins were normalized to β-actin, and data are presented as fold changes relative to the control group. All experiments were independently repeated three times (n = 3 biological replicates).

References

1. Cao Y, Zhao W, Zhong Y, Jiang X, Mei H, Chang Y*, et al.* Effects of chronic low-level lead (Pb) exposure on cognitive function and hippocampal neuronal ferroptosis: An integrative approach using bioinformatics analysis, machine learning, and experimental validation. **Sci Total Environ 2024**, 917**:** 170317.
2. Chang Y, Jiang X, Dou J, Xie R, Zhao W, Cao Y*, et al.* Investigating the potential risk of cadmium exposure on seizure severity and anxiety-like behaviors through the ferroptosis pathway in epileptic mice: An integrated multi-omics approach. **J Hazard Mater 2024**, 480**:** 135814.
3. Xie R, Xiao X, Zhao W, Zhong Y, Wu D, Dou J*, et al.* Association between long-term exposure of polystyrene microplastics and exacerbation of seizure symptoms: Evidence from multiple approaches. **Ecotoxicol Environ Saf 2025**, 302**:** 118741.
4. Dou J, Zhang K, Xie R, Xu H, Pan Q, Xiao X*, et al.* Investigating the Effects of Long-Term Fine Particulate Matter Exposure on Autism Spectrum Disorder Severity: Evidence from Multiple Analytical Approaches. **Toxics 2025**, 13(11).
5. Mei H, Wu D, Yong Z, Cao Y, Chang Y, Liang J*, et al.* PM(2.5) exposure exacerbates seizure symptoms and cognitive dysfunction by disrupting iron metabolism and the Nrf2-mediated ferroptosis pathway. **Sci Total Environ 2024**, 910**:** 168578.
6. Xie R, Zhao W, Lowe S, Bentley R, Hu G, Mei H*, et al.* Quercetin alleviates kainic acid-induced seizure by inhibiting the Nrf2-mediated ferroptosis pathway. **Free Radic Biol Med 2022**, 191**:** 212–226.
